# Control of carbendazim toxicity using banana peel powder in rats

**DOI:** 10.1016/j.btre.2022.e00773

**Published:** 2022-11-01

**Authors:** Gomaa N. Abdel-Rahman, Ahmed S.M. Fouzy, May M. Amer, Essam M. Saleh, Islam A. Hamed, Bassem A. Sabry

**Affiliations:** a Food Toxicology and Contaminants Department, National Research Centre, Dokki, Cairo, Egypt; b National Hepatology and Tropical Medicine Research Institute (NHTMRI), the Ministry of Health and Population, Egypt

**Keywords:** Carbendazim, Detoxification, Banana peels, Toxicity, Histopathology, Biochemical parameters

## Abstract

Carbendazim (CBZ) is a steady benzimidazole fungicide broadly exploited in cultivation for pre- and post-harvest treatment to dominate microorganisms infection on several plants. CBZ causes toxic effects in the different tissues of rat via influencing biochemical and hematological factors causing histopathological alterations in the liver and kidney of rats. Banana peel (BP) makes up about 38% of the whole banana weight, BP is usually disposed of as waste that is considered as an environmental problem. BP comprise bioactive a lot of compounds that can be exploited for their unique biological and pharmacological attributes. The current study was carried out to determine the protective effect of dried banana peels consumption against carbendazim toxicity in rats. The results indicated that banana peels had the ability to counteract the toxic effect of carbendazim on rats which was evident by the improvement in liver and kidney functions, lipid profile and histopathological examination.

## Highlights

• The use of banana peels may be safety as animal feed.
• The dried banana peels had protective effects against carbendazim toxicity.
• The dried banana peels may be used for reduction of toxic effects of pesticides on animals.

## 1 Introduction

Pesticides are manufactured compounds or biological active agents that are designed for drawing, attracting, destroying, or extenuating a lot of pest. They are mostly implemented in farming to defend crops from pests, wild plant, and microorganisms infections throughout plant development and to safeguard foods during storage from harmful animals, insects or bio-contaminants **[****1****]**. Some pesticides, like herbicides, are used to eliminate roadside pushes, trees, and shrubs and are usually used in ponds and lakes to prevent undesirable aquatic plants. Other pesticides are designed to avoid fungal growth or insects infestation in crops **[****2****]**. Thus, being a heterogeneous category, pesticides nowadays may be present almost all over the world. Pesticides residues from human activity can also reach water bodies via surface run- off, leaching, and/or erosion as noticed by Khan and Law **[****3****]**. Pesticides are specified by various grades of toxicity to different organisms **[****1****,** **3****]**. In the absence of regulation pesticides are broadly utilized might cause health risks to non-target organisms at numerous levels, involving those to human beings.

Carbendazim (CBZ), (Methyl-1H-Benzimidazol-2-ylcarbamate) is a steady benzimidazole fungicide broadly exploited in cultivation for pre- and post-harvest treatment to dominate microorganisms infection on several vegetables, fruits and different plants like banana, oranges, strawberries, pineapples, grains, sugar beet, ornamental plants and medical herbs **[****4****,** **5****]**. Nevertheless, steadiness and soil firmness can cause long-dated contamination, because its chemical construction supports adsorption inside the soil matrix and accumulation after repetitive applications **[****6****,** **7****]**. Additionally, the persistence of CBZ in soil changes the biodiversity of bacterial groups and negatively influences microbial functions **[****8****,** **9****]**. The half-life of CBZ varies from some days to 12 months depending on the type of the soil **[****8****]**.

CBZ caused toxic effects in the different tissues of rat via influencing biochemical and hematological factors causing histopathological alterations in the liver and kidney of rats **[****10****].** Persons may be exposed to CBZ through consumption of food commodities **[****6****].** CBZ is categorized in the harmful substances by World Health Organization and as possible human carcinogens **[****11****]**. CBZ has been prohibited in numerous countries because of its undesirable influences on the environment and health for example growth and reproductive disorders, toxicity and mutations **[****12****].**

Banana, scientifically known as *Musa sapientum*, is an herbaceous plant of the *Musaceae* family. The fruit is protected by its peel that is neglected as waste after eating the fleshy inner part **[****13****]**. Banana peel (BP) makes up more than 32% of the banana weight **[****14****]**. BP is usually disposed of as waste, which is an environmental problem as this by-product constitutes an environmental problem because it contains massive nitrogen and phosphorus quantities as well as its rich water content increase its susceptibility to microorganisms modifications. BP is rich in many biologically active chemicals including tannins, glycosides, terpenoids, flavonoids, and anthocyanins as reported by Pereira and Maraschin **[****15****].** They added that BP can be exploited for their unique biological and pharmacological properties such as antibacterial, hypotensive and anti-inflammatory**.**

Rebello et al. **[****16****]** and Sundaram et al. **[****17****]** declared that BP contains high content of micronutrients and antioxidants like polyphenols, prodolphenidine, carotenoids and catecholamines. That was also supported by Rattanvichai and Cheng **[****18****]** who reported that the BP provides beneficial pharmaceutical properties due to its content of bioactive chemicals. Moreover, gallocatechin was found to be five times higher in banana peel than in pulp, which suggests that banana peel is a rich source of antioxidants **[****19****]**. So, BP is one of the by-products that can be used as functional additives in the food industry **[****20****].** The current study was carried out to determine safety of banana peel as animal diet and the protective effect of dried banana peels consumption against carbendazim toxicity in rats.

## 2 Materials and methods

### 2.1 Banana fruits

The ripened fresh fruits of banana (*Musa*sp) were obtained from the farms of the Egyptian Ministry of Agriculture.

### 2.2 Preparation of banana peel

Fruits were checked for defects, insect damage, disease, surface color change and other defects to ensure the final product's quality. Banana fruits were washed thoroughly with distilled water to get rid of any dust or dirt that adheres to peel, and wiped dry. Fruit peels were separated manually and cut into small parts for about 2 × 2 cm, then dried using solar energy at 50 °C for 96 h. The banana peels were then ground thoroughly using mechanical grounding and passed through a 0.25 mm mesh.

### 2.3 Determination of active groups using fourier transform infrared spectra (FTIR)

Analysis of the functional groups present in the dried banana peel, as well as date stones, was carried out by absorption spectroscopy in the infrared region (400–4000 cm^−1^) at 4 cm^−1^ resolution **[****21****]**. The FTIR spectra were captured using a Bruker IFS48 spectrometer. To reduce spectrum contributions from ambient carbon dioxide and water vapor, the FTIR spectrometer was purged. The mean of four spectra from separate pellets of the same sample was then computed.

### 2.4 Experimental animals

This study is done according to the policies and ethical ways approved by the Animal Care and Use Committee of the National Research center, Dokki, Egypt. Twenty-Four **(**24) rats weighing 150–200 g. were obtained from the animal house in the National Research center. Rats were randomly divided into four (4) groups (*n* = 6/cage) in an environmentally controlled room 22 ± 2 °C/40–60% RH. Tap water and standard diet were available to rats. The acclimatization period was one week before the experiment.

### 2.5 Experimental design

Three of four test groups were treatment groups, one was control group (group 1) fed normal diet. The rats in the group (2) fed normal diet and additionally exposed to carbendazim fungicides (0.315 mg/ rat) oral dose. This dose was calculated as minimize of insecticides can affect on rats and the field application which equal 1/10 LD_10_ of insecticide. The rats in the group (3) fed normal diet with banana peel powders (20% of diet), this percentage was chosen according to our previous study **[****22****]**. The rats in the group (4) fed normal diet with banana peel powders (20% of diet) and additionally exposed to carbendazim fungicides (0.315 mg/ rat) oral dose. This experiment was achieved in the animal house -National Research center for 4 weeks. After the experimental period, blood samples are collected through retro-orbital venous plexus from each rat and centrifuged (4500 rpm/10 min) for separation of serum according to Cocchetto and Bjornsson **[****23****]**, and then the rats are sacrificed. The serum was stored in the deep freezer even analysis of liver function, kidney function and lipid profile.

### 2.6 Determination of biochemical parameters

The serum was separated and analyzed for liver function, kidney function and lipid profile using commercially diagnostic kits according to the manufacturer's instructions **[****24****]**. The levels of liver marker enzymes such as plasma aspartate amino transferase (AST), alanine amino transferase (ALT), alkaline phosphatase (ALK-P) and gamma-glutamyl transferase (GGT) were determined. Also, the levels of kidney function (urea, creatinine and uric acid) as well as lipid profile (triglycerides, total cholesterol, HDL cholesterol and LDL cholesterol) were examined.

### 2.7 Histopathological studies

The animals were sacrificed and organs of liver and kidney were collected in falcon tubes contain 10% neutral buffered formalin, then the tissue slides are stained with hematoxylin and eosin for histopathological examination **[****25****]**.

### 2.8 Statistical analysis

Results were subjected to one-way analysis of variance (ANOVA) of the general linear model (GLM) using SAS **[****26****]** statistical package. The results were the average of three experiments (*p* ≤ 0.05).

## 3 Results and discussion

### 3.1 Active groups and characterizations of banana peel

**Fig. 1:**
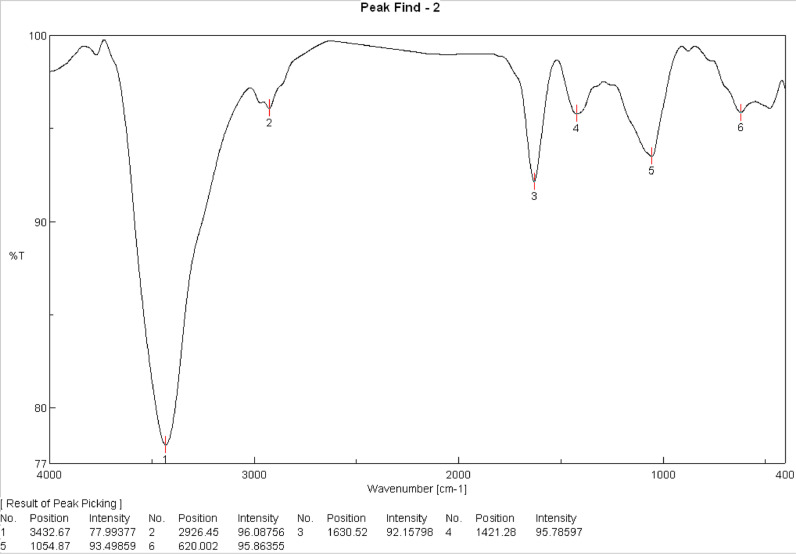
FTIR spectra of banana peel.

Fourier Transform Infrared spectra (FTIR) of were used to understand the nature of the functional groups in banana peels. Data in Figure (1) displayed several peaks for the banana peels. Bands appeared at 3432.67, 2926.45, 1630.5, 1421.28, 1054.87, and 620.002 cm^−1^ were assigned to O—H stretching, C—H stretching of alkane (stretching of carboxylic acid or ester), C=C stretching of alkene, and -C-H bending of alkane, respectively. Therefore, the FTIR spectrum profile established the occurrence of carboxylic acid, alcohol, alkenes and amines. Similar results were reported by Pathak *et al*. **[****27****]**. Out of these functional groups, carboxylic acid and hydroxyl groups may have played a principal role in the removal of pesticides **[****28****]**. The main source of carboxylic acid in fruit peel could be cellulose, pectin, or lignin **[****29****]**. On the other hand, the FTIR did not show any peaks between 2220 and 2260 cm^–1^, thus suggesting the absence of cyanide groups, and confirming that banana peel does not contain any toxic substances **[****30****]**(Fig. 1).

### 3.2 Biochemical parameters

The concentrations of liver marker enzymes such as serum aspartate amino transferase (AST), alanine amino transferase (ALT), alkaline phosphatase (ALK-P) and gamma-glutamyl transferase (GGT) in rat's serum were determined and recorded in Table (1). The results revealed that, there were a significant (*p* < 0.05) increases in the ALT, AST and ALK-P among the experimental rat's dosed carbendazim (group 2) when compared with the control group (group 1) by 14.1, 10.4 and 13.9%, respectively, while no significant increase in the level of serum GGT. An increase in levels of AST, ALT and ALK-P may indicate hepatocellular disease, active cirrhosis and metastatic liver. The rat's diet with 20% banana peels and dosed carbendazim (group 4) showed significant decrease of ALT and ALK-P levels in rat's serum comparing with the carbendazim group (group 2) by 7.3 and 6.4%, respectively, while doesn't have any effect on AST levels. These results suggest that the banana peels had a possible protective effect against carbendazim toxicity on liver functions.

**Table 1:** Effect of banana peels (20%) on some serum liver marker enzymes concentration in rats oral with carbendazim (0.315 mg/ rat).

Experimental groups	Liver functions (U/L)			
	Mean ± S*.*E.			
	ALT-SGPT	AST-SGOT	ALK-P	GGT
Group (1) control	145.7 ^bc^ ± 4.6	156.0 *^b^* ± 3.4	156. 7 *^b^* ± 3.5	72.5 *^a^* ± 2.6
Group (2) dosed carbendazim	166.3 *^a^* ± 4.3	172.3 *^a^* ± 6.4	178.5 *^a^* ± 2.8	77.7 *^a^* ± 2.0
Group (3) 20% banana peel	140.5 *^c^* ± 3.9	156.0 *^b^* ± 3.7	157.5 *^b^* ± 3.9	71.8 *^a^* ± 2.1
Group (4) 20% banana peel and dosed carbendazim	154.2 *^b^* ± 1.8	165.2 ^ab^ ± 5.4	167.0 *^b^* ± 4.1	74.8 *^a^* ± 1.9
LSD	11.3	14.4	10.6	6.4

Means followed by different subscripts within column are significantly different at the 5% level.

LSD: Least Significant Difference.

The results revealed mild hepatotoxicity due to carbendazim ingestion manifested by the elevation in the tested liver function indicating some destruction that occur in liver cell, that degeneration could be attributed to free radicals generated due to carbendazim. Treatment with banana peels showed significant improvement in all the tested liver parameters and since banana peels contain vitamins (C, E, and B6), phenols, minerals (K, Ca, Na, Fe and Mn), essential amino acids (leucine-valine-phenylalanine and threonine) and polyunsaturated fatty-acids (linoleic acid) which have antioxidant properties that supports as protective agents for the body against diseases and free radicals **[****31****]**. They added that the banana peels had significant decrease in liver function of rats injected by carbon tetrachloride (to induce liver failure) comparing with the positive control group. Also, these results are in harmony with the study of Abdel-Rahman *et al*. **[****32****]** who found that the banana peel had a practical role in detoxification of aflatoxins in albino rats due to decrease the levels of AST, ALT, ALK-P in the serum.

Regarding the evaluation of some serum kidney marker enzymes concentration in rats, the results in Table (2) revealed that administration of carbendazim (0.315 mg/ rat) although did not record significant change in either urea or uric acid yet there was a significant increase in creatinine level to 0.72 ± 0.06 which in turn is an indication of kidney dysfunction due to nephrotoxicity of carbendazim exposure. Our findings on CBZ-nephrotoxicity are in accordance with previous results reported by Selmanoglu *et al*. **[****6****]**, Abolaji *et al*. **[****33****]** and Owumi *et al*. **[****34****].** An insignificant increment of serum urea level is usually accompanied with decreased reabsorption at the renal epithelium, whereas serum creatinine elevation refers to impairment in the kidney function especially in the glomerular filtration rate **[****35****]**. The group of rats treated with 20% banana peels showed marked improvement as the creatinine level decreased to 0.62±0.03 reversing toward the normal levels. This observation is agree with Nagib and Ataya **[****36****]** who found that the dried banana peels had great therapeutic effects against lead toxicity and improvements liver enzyme activities and kidney function. The protective effect of banana peel powder against carbendazim toxicity may be return to the presence of functional groups such as carboxylic acid, alcohol, alkanes and amines (Fig. 1).

**Table 2:** Effect of banana peels (20%) on some serum kidney marker enzymes concentration in rats oral with carbendazim (0.315 mg/ rat).

Experimental groups	kidney functions (mg/d.l)		
	Mean ± S*.*E.		
	Urea	Creatinine	Uric acid
Group (1) control	19.5 *^b^* ± 0.9	0.45 *^b^* ± 0.04	1.50 *^b^* ± 0.07
Group (2) dosed carbendazim	21.0 *^b^* ± 1.0	0.72 *^a^* ± 0.06	1.56 ^ab^ ± 0.07
Group (3) 20% banana peel	28.8 *^a^* ± 1.4	0.49 *^b^* ± 0.03	1.72 ^ab^ ± 0.08
Group (4) 20% banana peel and dosed carbendazim	29.3 *^a^* ± 1.4	0.62 *^a^* ± 0.03	1.77 *^a^* ± 0.08
LSD	3.5	0.12	0.22

Means followed by different subscripts within column are significantly different at the 5% level.

LSD: Least Significant Difference.

Data in Table (3) reveals an obvious disturbance in the lipid profile as there is non-significant elevation in the triglycerides in the rats group receiving carbendazim this elevation was reduced significantly in the two groups of rats receiving banana peel indicating the improvement in liver performance. The same observation was recorded for the levels of cholesterol levels which increased non-significantly in the rats group receiving carbendazim and significant reduction was recorded in the rats receiving banana peel. Our results on carbendazim hepatotoxicity support previous findings **[****37****]**.

**Table 3:** Effect of banana peels (20%) on lipids profile in rats oral with carbendazim (0.315 mg/ rat).

Experimental groups	Lipids profile			
	Mean ± S*.*E.			
	Triglycerides (mg/d.l)	Cholesterol (mg/d.l)	HDL cholesterol (mg/d.l)	LDL cholesterol (mg/d.l)
Group (1) control	129.0 *^a^* ± 3.3	77.2 ^ab^ ± 5.0	27.2 *^a^* ± 1.1	49.5 *^a^* ± 1.4
Group (2) dosed carbendazim	138.0 *^a^* ± 4.3	87.8 *^a^* ± 4.2	24.2 ^ab^ ± 1.4	43.0 *^b^* ± 2.0
Group (3) 20% banana peel	105.3 *^b^* ± 4.4	70.0 *^b^* ± 2.8	21.5 ^bc^ ± 0.9	41.3 *^b^* ± 1.2
Group (4) 20% banana peel and dosed carbendazim	112.0 *^b^* ± 3.1	72.0 *^b^* ± 2.4	20.2 *^c^* ± 0.7	45.3 ^ab^ ± 1.4
LSD	11.7	11.2	3.2	4.8

Means followed by different subscripts within column are significantly different at the 5% level.

LSD: Least Significant Difference.

The main phenolic compounds in the banana peel were flavonols, hydroxycinnamic acids, flavan-3-ols and catecholamines **[****38****]**. Also, banana peel contain dietary leucine that have many of health benefits such as hyperglycaemia, hypercholesterolemia and reduction in diet-induced weight gain **[****39****].** In addition, banana peel ameliorate the lipid profile could be return to the high levels of Mg ions and dietary fiber. Mg ion regulates a specific enzyme (HMG-CoA reductase), a rate limiting step of cholesterol synthesis in the body **[****40****].** In addition, Mg ion is needful for activity of lecithin cholesterol acyl transferase (LCAT), which reduce the levels of LDL-C and triglyceride, while increase the levels of raises HDL-C **[****41****].**

### 3.3 Histopathological investigations of kidney

**Fig. 2:**
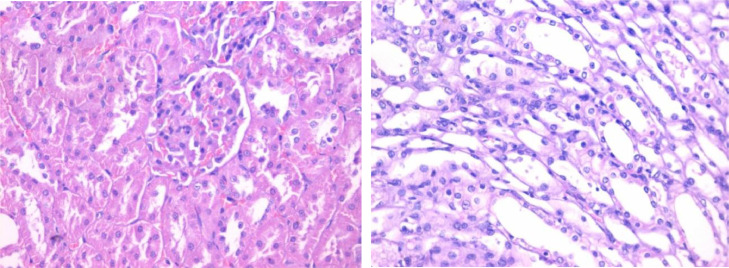
Section in kidney tissue of group (1): showing normal kidney tissue (Hematoxylin and eosin stain, X400).

**Fig. 3:**
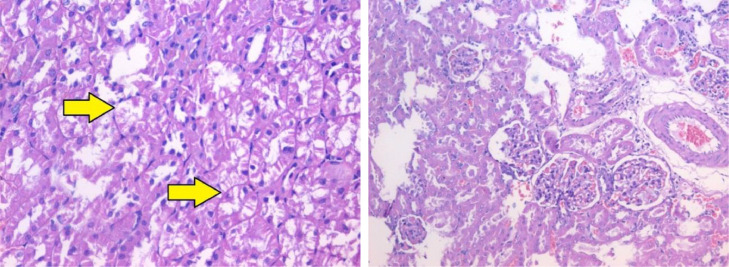
Section in kidney tissue of group (2): showing focal tubal epithelial degeneration (yellow arrow) (Hematoxylin and eosin stain, X400).

**Fig. 4:**
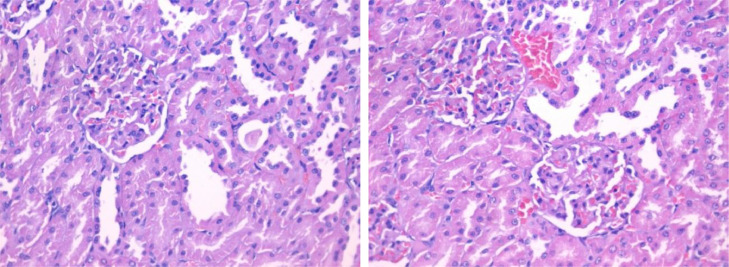
Section in kidney tissue of group (3): showing no obvious histopathological abnormality at the light microscopic level (Hematoxylin and eosin stain, X400).

**Fig. 5:**
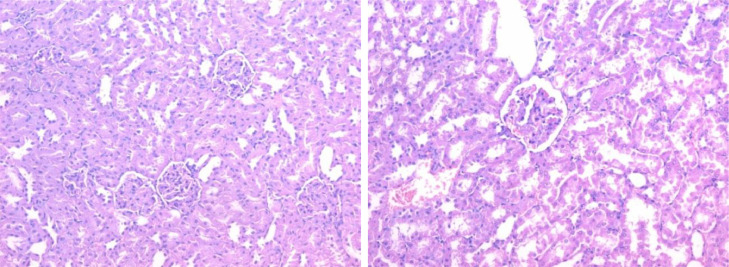
Section in kidney tissue of group (4): showing no obvious histopathological abnormality at the light microscopic level (Hematoxylin and eosin stain, X400).

The histopathological lesion scoring in kidney of mice in the different experimental groups was summarized in the Table (4) and Figures (2–5). Normal histological architecture was seen in the renal tissue of normal control mice (Fig. 2). Meanwhile, severe histopathological alterations were noticed in the renal parenchyma of carbendazim treated mice which represented by marked vacuolations of renal tubular epithelium, pyknosis of nuclei (Fig. 3). These observations are conformable with Selmanoglu *et al*. **[****6****]** who noticed that expose of rats to 300 and 600 mg kg^−1^ per day carbendazim led to fibrosis, congestion, mononuclear cell infiltration and tubular degeneration in the kidney. Also, the kidney of rats exposed to carbendazim (50 mg/kg) showed focal area of necrosis and the presence of inflammatory cells as reported by Owumi *et al*. **[****34****]**.

**Table 4:** Histopathological alterations in Kidney and liver tissues.

Group	Liver			Kidney		
	Hep. Degeneration	Apoptosis	Inflammation	Others	Glomeruli	Tubules
G1	±*N*	–	–	–	±*N*	±*N*
G2	+	+	±*N*	Kupffer cell hyperplasia +	±*N*	Tubal deg. +
G3	±*N*	–	+	–	±*N*	±*N*
G4	±*N*	–	Portal +	±	±*N*	±*N*

Otherwise, renal sections of mice treated with banana peel no histopathological alterations with slight congestion of inter-tubular blood vessels (Fig. 4). On the other hand, kidney tissue of rats treated with carbendazim + banana peel powder exhibited marked restoration of the histological structure (Fig. 5). The protective effect of banana peel powder against histopathological alterations of carbendazim may be return to the antioxidants such as polyphenols, prodolphenidine, carotenoids and catecholamines which found in the banana peels **[****16****,** **17****]**. Furthermore, Hikal *et al*. **[****42****]** demonstrated that the banana peel contains many important phytochemicals and offers many health benefits. Also, Kamal *et al*. **[****43****]** reported that the banana peels extract had anti-cancer agents, radioprotective, improving hematological parameters and attenuated lipid peroxidation of mice.

### 3.4 Histopathological investigations of liver

**Fig. 6:**
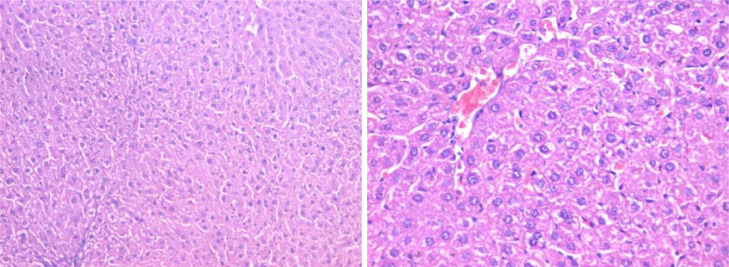
Section in liver tissue of group (1): showing normal hepatic architecture (Hematoxylin and eosin stain, X400).

**Fig. 7:**
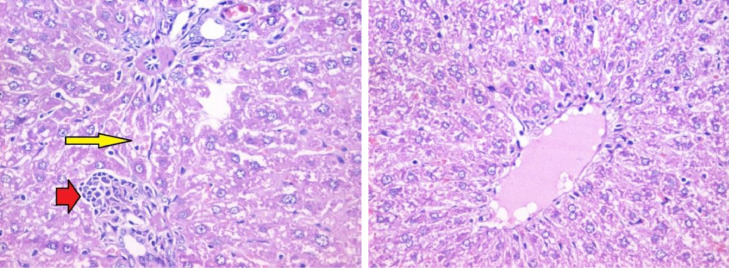
Section in liver tissue of group (2): focal infiltration of the portal tract by mononuclear inflammatory cells (red arrow) and focal hepatocellular degeneration (yellow arrow) (Hematoxylin and eosin stain, X400).

**Fig. 8:**
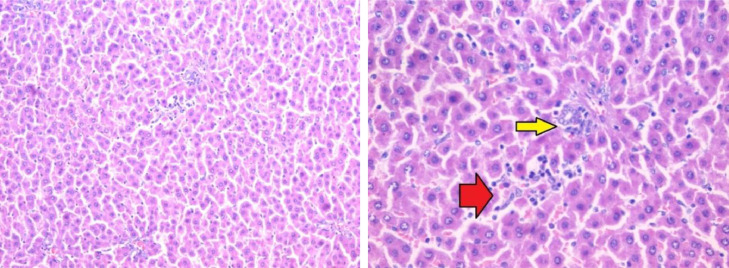
Section in liver tissue of group (3): showing focal infiltration of the hepatic lobule (red arrow) and portal tract (yellow arrow) by mononuclear inflammatory cells (yellow arrows) (Hematoxylin and eosin stain, X400).

**Fig. 9:**
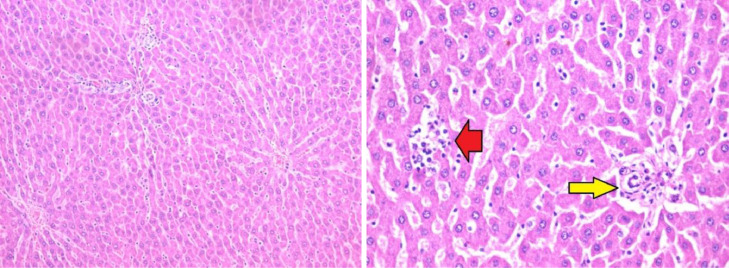
Section in liver tissue of group (4) showing focal infiltration of the hepatic lobule (red arrow) and portal tract (yellow arrow) by mononuclear inflammatory cells (yellow arrows) (Hematoxylin and eosin stain, X400).

The histopathological lesion scoring in liver of mice in the different experimental groups was summarized in the Table (4) and Figures (6–9). Microscopic examination of liver of untreated rats showed the normal architecture of hepatic parenchyma that consists of central veins and hepatocytes arranged in hepatic cords (Fig. 6). In contrast, liver of rats from group 2 (administered carbendazim only) exhibited remarkable histopathological alterations characterized by Kupffer cells proliferation, hepatocellular steatosis with karyomegaly of some hepatocytic nuclei and apoptosis (Fig. 7). Furthermore, fibroplasia and portal infiltration with mononuclear cells were also noticed. These results are in agreement with those obtained by Selmanoglu *et al*. **[****6****]** who observed that the number of Kupffer cells and mononuclear cell infiltration were increased in the liver of rats treated with 150, 300 and 600 mg kg^−1^ per day carbendazim. Also, the added that the congestion in portal veins and an enlargement of the sinusoids were recorded in the liver of treated rats. In addition, Owumi *et al*. **[****34****]** mentioned that the liver of rats administered with 50 mg/kg of carbendazim had severe disseminated congestion and infiltration of inflammatory cells.

Meanwhile, liver of rat's diet with banana peel powder exhibited no histopathological lesions (Fig. 8). On the other hand, hepatic tissue of rats treated with carbendazim + banana peel powder revealed moderate improved changes, liver sections showed slight hepatocyte inflammation and focal hepatocellular steatosis (Fig. 9). Moreover, the hepatic parenchyma of carbendazim rats treated with banana peel exhibited marked restoration of the histological structure, examined sections described except slight degeneration of some hepatocytes. The protective effect of banana peel powder against carbendazim toxicity may be return to the presence of vitamin E which has antioxidant properties **[****31****]**. This observation is accordance with Rajeswary *et al*. **[****44****]** who found that the various histopathological changes were absents in the tissue of rats treated with carbendazim + vitamin E.

## 4 Conclusion

The previous results demonstrated the high toxicological impact of carbendazim exposure on the biochemical and histopathological parameters and indicated the possible use of banana peel powder as a protective adjutant in experimental animals.

## Declaration of Competing Interest

The authors have no conflict of interest

## References

[1] Bolognesi C., Merlo F.D., Nriagu J.O. (2011). Encyclopedia of Environmental Health.

[2] Gupta P.K., Gupta R.C. (2011). Reproductive and Developmental Toxicology.

[3] Khan Z.M., Law F.C.P. (2005). Adverse effects of pesticidesand related chemicals on enzyme and hormone systems of fish, amphibians and reptiles: a review. Proc. Pakistan Acad. Sci..

[4] Tortella G.R., Mella-Herrera R.A., Sousa D.Z., Rubilar O., Briceno G., Parra L., Diez M.C. (2013). Carbendazim dissipation in the biomixture of on-farm biopurification systems and its effect on microbial communities. Chemosphere.

[5] Devi P.A., Paramasivam M., Prakasam V. (2015). Degradation pattern and risk assessment of carbendazim and mancozeb in mango fruits. Environ. Monit. Assess..

[6] Selmanoglu G., Barlas N., Songur S., Kockaya E.A. (2001). Carbendazim-induced haematological, biochemical and histopathological changes to the liver and kidney of male rats. Hum. Experiment. Toxicol..

[7] Lewandowska A., Walorczyk S. (2010). Carbendazim residues in the soil and their bioavailability to plants in four successive harvests. Pol. J. Environ. Stud..

[8] Singh S., Singh N., Kumar V., Datta S., Wani A.B., Singh D., Singh K., Singh J (2016). Toxicity, monitoring and biodegradation of the fungicide carbendazim. Environ. Chem. Lett..

[9] Wang L., Wang B., Li H., Lu H., Qiu F., Xiong L., Xu Y., Wang G., Liu X., Wu H., Jing H. (2012). Quercetin, a flavonoid with anti-inflammatory activity, suppresses the development of abdominal aortic aneurysms in mice. Eur. J. Pharmacol..

[10] Veerappan M., Pandurangan M., Suriyamurthy M., Kotteazeth S. (2011). Acute toxicological evaluation of low dose methyl 2-benzimidazole carbamate fungicide on male Albino rats. Iranian J. Toxicol..

[11] Goodson W.H., Lowe L., Carpenter D.O., Gilbertson M., Ali A.M., de Cerain-Salsamendi A.L. (2015). Assessing the carcinogenic potential of low-dose exposures to chemical mixtures in the environment: the challenge ahead. Carcinogenesis.

[12] Costa E.P.da, Bottrel S.E.C., Starling M.M.D.Leao, Amorim C.C. (2018). Degradation of carbendazim in water via photo-fenton in raceway pond reactor: assessment of acute toxicity and transformation products. Environ. Sci. Pollut. Res..

[13] Anhwange B.A. (2008). Chemical composition of Musa sapientum (Banana) peels. J. Food Technol..

[14] Gonzalez-Montelongo R., Gloria L.M., Gonzalez M. (2010). Antioxidant activity in banana peel extracts: testing extraction conditions and related bioactive compounds. Food Chem..

[15] Pereira A., Marschin M. (2015). Banana (Musa spp) from peel to pulp: ethno pharmacology, source of bioactive compounds and its relevance for human health. J. Ethnopharmacol..

[16] Rebello L.P.G., Ramos A.M., Pertuzatti P.B., Barcia M.T., Castillo-Munoz N., Hermosín-Gutiérrez I. (2014). Flour of banana (Musa AAA) peel as a source of antioxidant phenolic compounds. Food Res. Int..

[17] Sundaram S., Anjum S., Dwivedi P., Rai G.K. (2011). Antioxidant activity and protective effect of banana peel against oxidative hemolysis of human erythrocyte at different stage of ripening. Appl. Biochem. Biotechnol..

[18] Rattanvichai W., Cheng W. (2015). Dietary supplement of banana (Musa acuminate) peels hot-water extract to enhance the growth, anti-hypothermal stress, immunity and disease resistance of the giant freshwater prawn, Macrobrachium rosenbergii. Fish Shellfish Immunol..

[19] Someya S., Yoshiki Y., Okubo K. (2002). Antioxidant compounds from bananas (Musa Cavendish). Food Chem..

[20] Hana B.M.Z., Mohd D.B.S., Wolyna P. (2020). The roles of banana peel powders to alter technological functionality, sensory and nutritional quality of chicken sausage. Food Sci. Nutr..

[21] Vicentini N.M., Dupuy N., Leitzelman M., Cereda M.P., Sobral P.J.A. (2005). Prediction of cassava starch edible film properties by chemometric analysis of infrared spectra. Spectrosc. Lett..

[22] Sanad M.H., Abdel Rahim E.A., Hathout A.S., Hussain O.A., Rashed M.M., Fouzy A.S.M. (2022). Distribution of iodine125 labeled parathion and the protective effect of dried banana peel in experimental mice. Egypt. J. Chem..

[23] Cocchetto D.M., Bjornsson T.D. (1983). Methods for vascular access and collection of body fluids from the laboratory rat. J. Pharm. Sci..

[24] Hasan K.M.M., Tamanna N., Haque M.A. (2018). Biochemical and histopathological profiling of Wistar rat treated with Brassica napus as a supplementary feed. Food Sci. Hum. Wellness.

[25] Suvarna K., Layton C., Bancroft J (2012).

[26] SAS (1999). Release 6.03 Edn.

[27] Pathak P.D., Mandavgane S.A., Kulkarni B.D. (2017). Fruit peel waste: characterization and its potential uses. Curr. Sci..

[28] Memon J.R., Memon S.Q., Bhanger M.I., Memon G.Z., El-Turki A., Allen G.C. (2008). Characterization of banana peel by scanning electron microscopy and FT-IR spectroscopy and its use for cadmium removal. Coll. Surf. B: Biointerf..

[29] Schiewer S., Patil S.B. (2008). Pectin-rich fruit wastes as biosorbents for heavy metal removal: equilibrium and kinetics. Bioresour. Technol..

[30] Mohapatra D., Mishra S., Sutar N. (2010). Banana and its by-product utilization: an overview. J. Sci. Ind. Res. (India).

[31] Mosa Z.M., Khalil A.F. (2015). The effect of banana peels supplemented diet on acute liver failure rats. Ann. Agric. Sci..

[32] Abdel-Rahman T.M., Ali D.M.I., Abo-Hagger A.A., Ahmed M.S. (2017). Efficacy of banana peel in reduction of aflatoxin toxicity in rats. J. Agric. Chem. and Biotechn., Mansoura Univ..

[33] Abolaji A.O., Awogbindin I.O., Adedara I.A. (2017). Insecticide chlorpyrifos and fungicide carbendazim, common food contaminants mixture, induce hepatic, renal, and splenic oxidative damage in female rats. Hum. Exp. Toxicol..

[34] Owumi S.E., Nwozo S.O., Najophe E.S. (2019). Quercetin abates induction of hepatic and renal oxidative damage, inflammation, and apoptosis in carbendazim-treated rats. Toxicol. Res. Appl..

[35] Adedara I.A., Teberen R., Ebokaiwe A.P. (2012). Induction of oxidative stress in liver and kidney of rats exposed to Nigerian bonny light crude oil. Environ. Toxicol..

[36] Nagib E.W., Ataya H.R. (2021). Effect of mango and banana peels induced on toxicity by lead acetate in rats. Home Econ. J..

[37] Muthuviveganandavel V., Muthuraman P., Muthu S., Srikumar K. (2008). Toxic effects of carbendazim at low dose levels in male rats. J. Toxicol. Sci..

[38] Zaini H.M., Roslan J., Saallah S., Munsu E., Sulaiman N.S., Pindi W. (2022). Banana peels as a bioactive ingredient and its potential application in the food industry. J. Funct. Foods.

[39] Zhang Y., Guo K., LeBlanc R.E., Loh D., Schwartz G.J., Yu Y.H. (2007). Increasing dietary leucine intake reduces diet-induced obesity and improves glucose and cholesterol metabolism in mice via multimechanisms. Diabetes.

[40] Son C., Hosoda K., Ishihara K., Bevilacqua L., Masuzaki H., Fushiki T., Harper M.E., Nakao K. (2004). Reduction of diet-induced obesity in transgenic mice overexpressing uncoupling protein 3 in skeletal muscle. Diabetologia.

[41] Kumar K.S., Bhowmik D., Duraivel S., Umadevi M. (2012). Traditional and medicinal uses of banana. J. Pharmacogn. Phytochem..

[42] Hikal W.M., Kacaniova M., Said-Al Ahl H.A.H. (2021). Banana Peels as Possible Antioxidant and Antimicrobial Agents. Asian J. Res. Review in. Agricult..

[43] Kamal A.M., Taha M.S., Mousa A.M. (2019). The radioprotective and anticancer effects of banana peels extract on male mice. J. Food Nutrit. Res..

[44] Rajeswary S., Mathew N., Akbarsha M.A., Kalyanasundram M., Kumaran B. (2007). Protective effect of vitamin E against carbendazim-induced testicular toxicity–histopathological evidences and reduced residue levels in testis and serum. Arch. Toxicol..

